# Abstract art and cortical motor activation: an EEG study

**DOI:** 10.3389/fnhum.2012.00311

**Published:** 2012-11-16

**Authors:** M. Alessandra Umilta', Cristina Berchio, Mariateresa Sestito, David Freedberg, Vittorio Gallese

**Affiliations:** ^1^Department of Neuroscience, Section of Physiology, University of ParmaParma, Italy; ^2^Department of Art History and Archaeology, Columbia UniversityNew York, NY, USA

**Keywords:** cortical motor system, perception, abstract art, EEG, mu rhythm suppression

## Abstract

The role of the motor system in the perception of visual art remains to be better understood. Earlier studies on the visual perception of abstract art (from Gestalt theory, as in Arnheim, 1954 and 1988, to balance preference studies as in Locher and Stappers, 2002, and more recent work by Locher et al., 2007; Redies, 2007, and Taylor et al., 2011), neglected the question, while the field of neuroesthetics (Ramachandran and Hirstein, 1999; Zeki, 1999) mostly concentrated on figurative works. Much recent work has demonstrated the multimodality of vision, encompassing the activation of motor, somatosensory, and viscero-motor brain regions. The present study investigated whether the observation of high-resolution digitized static images of abstract paintings by Lucio Fontana is associated with specific cortical motor activation in the beholder's brain. Mu rhythm suppression was evoked by the observation of original art works but not by control stimuli (as in the case of graphically modified versions of these works). Most interestingly, previous visual exposure to the stimuli did not affect the mu rhythm suppression induced by their observation. The present results clearly show the involvement of the cortical motor system in the viewing of static abstract art works.

## Introduction

It is now established that the observation of goal-related motor acts or of the movement of body parts leads to the activation of their cortical motor representations in observers' brains (for review, Keysers and Gazzola, 2009; Rizzolatti and Sinigaglia, 2010). It has been shown that static images of goal-related hand-object interactions (Johnson-Frey et al., 2003) or of body movements and actions (Mado-Proverbio et al., 2009) also induces motor activation in observers' brains. Furthermore, several studies show that cortical motor activation can be induced when the observed stimuli are static graphic artifacts produced by hand motor acts, such as a letters. Longcamp et al. (2006) used magnetoencephalography (MEG) to study the modulation of 20 Hz oscillations in the hand representation of the primary motor cortex during observation of letters. A suppression of the oscillations was shown both during hand movements and the observation of static letters, the modulation effect being stronger for the observation of handwritten than of typed letters. This evidence suggests that the activation of the cortical motor system can be evoked by observing the static graphic outcome of an agent's action. It could thus be hypothesized that the activation of the cortical motor system might be relevant during the observation of particular static objects like abstract works of art.

The relatively new field of neuroesthetics (e.g., Ramachandran and Hirstein, 1999; Zeki, 1999; for review, see Di Dio and Gallese, 2009) has begun to examine the neural basis of the observation of works of art. So far, most of this research has conceived of the perception of works of art merely in visual terms. The role of the motor system in the observation of visual art has been much neglected. But the complexity of the relation between an artwork and its observer goes well beyond the ability of the brain to capture essential perceptual elements from observed objects by vision alone. Vision is a multimodal enterprise, encompassing the activation of motor, somatosensory, and viscero-motor brain regions (Gallese and Di Dio, 2012). It was recently proposed that the visible traces of the artist's creative gestures, like brush strokes or cuts on the canvas, are the visible traces of goal-directed movements, and that in principle they should be capable of activating the relevant motor areas in the observers' brain (Freedberg and Gallese, 2007).

The aim of the present study was to investigate whether the visual perception of static images from abstract art works might be associated with specific cortical motor activation in their perceivers. Using high-density electroencephalography (EEG) we measured the intensity of mu rhythm suppression from the cortical central areas during the observation of high-resolution digitized images of abstract paintings by Lucio Fontana and of graphically modified versions of them.

## Methods

### Participants

Fourteen healthy volunteers (7 males, 7 females, mean age 28.3 years; SD ± 4.2) participated in the experiment. Participants were recruited by public announcement and were blind to the experimental goals. All participants were right-handed as assessed by the Edinburgh Handedness Inventory (Oldfield, 1971). None of them reported the presence of any neurological or psychiatric disorder and had normal or corrected to normal vision. Before the experiment, all participants received experimental instructions. Written informed consent was obtained from all participants before entering the study. The Ethical Committee of the University of Parma approved the study.

### Experimental procedure

Participants were seated comfortably in front of a 17-inch computer monitor used for stimuli presentation, located at a distance of 60 cm. To minimize participants' movements during the experiment, they were asked to keep their arms on a table in front of them and to stay as motionless and relaxed as possible. During the experiment participants passively observed the presented images and were asked to pay attention to them.

**Figure 1:**
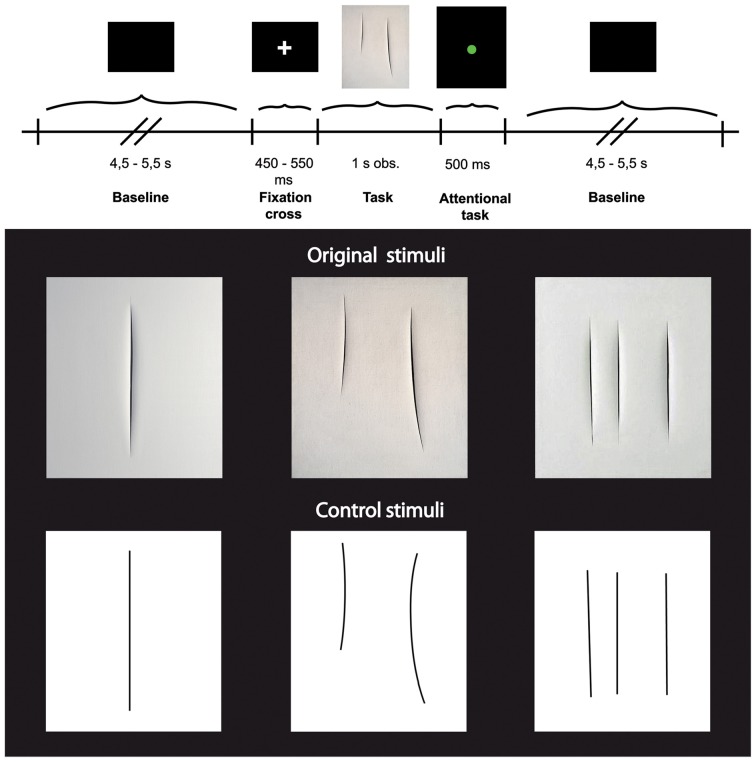
**Upper panel:** Experimental paradigm. **Lower panel:** Photographs of three Original art works by Lucio Fontana (top) and three graphically modified version of them (Control stimuli, bottom).

Each trial started with a black background (baseline) with a varying duration of 4.5–5.5 s (±500 ms, three possible times: 4500, 5000, or 5500 ms), followed by a single fixation cross presented for a period from 450–550 ms (±50 ms, three possible times: 450, 500, or 550 ms**)**, and then by the visual presentation of the stimuli for 1 s (Figure 1 upper panel). In half of the trials, the stimulus display was followed by the presentation of a colored circle for 500 ms. The circles could be red or green and participants were requested to give a verbal response as to the color of each circle. This task was unrelated to the aim of the experiment and was introduced to keep participants' attention constant. In order to avoid possible overlap and/or interference between the baseline and the task, in particular during the trials in which participants were asked to give the verbal response, the first 2 s of the baseline were always excluded from subsequent analyses (see also below: EEG data analysis).

Stimuli consisted of two categories of images (see Figure 1 lower panel) showing: (1) 3 different black and white high-resolution digitized images of abstract artworks of Lucio Fontana (Original Stimuli, Original condition) showing one, two, and three cuts on the canvas; (2) 3 high-resolution digitized images of graphically modified versions of the original artworks (Control Stimuli, Control condition) displaying the same black and white graphic pattern of the original images. All stimuli (total of 6) were randomly presented, 15 times each for a total number of 90 stimuli. The 3 digitized images of art works (Original stimuli) have been downloaded from open source web sites.

The control stimuli were created by means of Adobe Photoshop CS5.1 software, using the original artworks as template. We overlapped a transparent grid on the original images and replaced cuts with lines of the same length and thickness.

### Rating of the stimuli

At the end of the recording session, after removing the net from the head, all stimuli were randomly showed once again and each participant was asked to score for each stimulus its: (1) Familiarity (“If and how are you familiar with this image,” score from 0 to +10); (2) Aesthetic appraisal (“How much do you like this image?,” score from −10 to +10); (3) Amount of movement (“If and how much movement do you perceive in this image,” score from 0 to +10); (4) Artistic nature of the perceived images (“Is the image a real artwork?,” score 1 if the answer was “yes” and 0 if the answer was “no”).

Participants were divided in two groups (Familiar and Unfamiliar) on the basis of the average of the score that each of them gave to the 3 Original stimuli in the Familiarity score. When the Familiarity score was <3 the participant was considered unfamiliar. When the Familiarity score was ≥3 the participant was considered familiar. The average score of the Unfamiliar group was 0.71 (SE ± 0.42), the average score of the Familiar group was 7.6 (SE ± 0.6). The restrictive cut off score of 3 was chosen in order to include in the Familiar group the participants that were even slightly familiar with Original artworks of Lucio Fontana. The Familiar group comprised 5 males and 2 females while the Unfamilar group was composed of 5 females and 2 males.

### EEG recording

EEG data were acquired by a 128-channel Sensor Net (Electrical Geodesic, Eugene, OR, USA) and recorded within standard EGI package Net Station 4.3.1. EEG was sampled at 250 Hz and band-pass filtered at 0.3–100 Hz. Electrodes impedance was less than 50 KΩ. Raw EEG data were recorded with the vertex (Cz) as the online reference and re-referenced off-line to the common average (Muthukumaraswamy et al., 2004).

Stimuli were presented with E-Prime 2.0. and all event markers were sent to Net Station. The experiment took place in an isolated and lit room. Participants' motion was monitored by the experimenter and video-recorded for off-line analysis; if participants moved during the observation or rest conditions, the trial was excluded from further data analysis.

### EEG data analysis

EEG data were filtered off-line with band-pass filter 0.3–30 Hz and segmented into two time epochs. One thousand ms of black screen before the appearance of the fixation cross were taken as baseline. In particular, the central period from 2000 to 3000 ms after baseline onset was selected as baseline for further analysis (Baseline condition). Stimuli presentation lasted 1000 ms and was considered the experimental period. The baseline and stimuli presentation epochs were taken for the wavelet transformation (see below). The trials in which participants produced eye blinks and movement artifacts were rejected. Artifacts detection has been performed with different criteria: (1) eyes blinks and bad channels were detected by default parameters set by the software (see below); (2) any artifacts was further detected by careful visual inspection of the EEG traces. The default parameters for rejection were the following: bad channels: max–min >200 μV, eye blink: max–min >140 μV, eye movements: max–min >50 μV. Seventy-three point seventy-three percent (73.73%) (SE ± 4.3) of trials in the Original condition and 73.09 % (SE ± 4.1) of trials in the Control condition were kept for the statistical analyses.

The time-frequency analysis was performed by continuous Morlet wavelet transformation in 0.5 Hz intervals in the frequency range from 1 to 30 Hz. Wavelet coefficients squared as a function of frequency were calculated by taking the average across trials. The wavelet transformation was calculated separately for each participant in all 128 channels.

**Figure 2:**
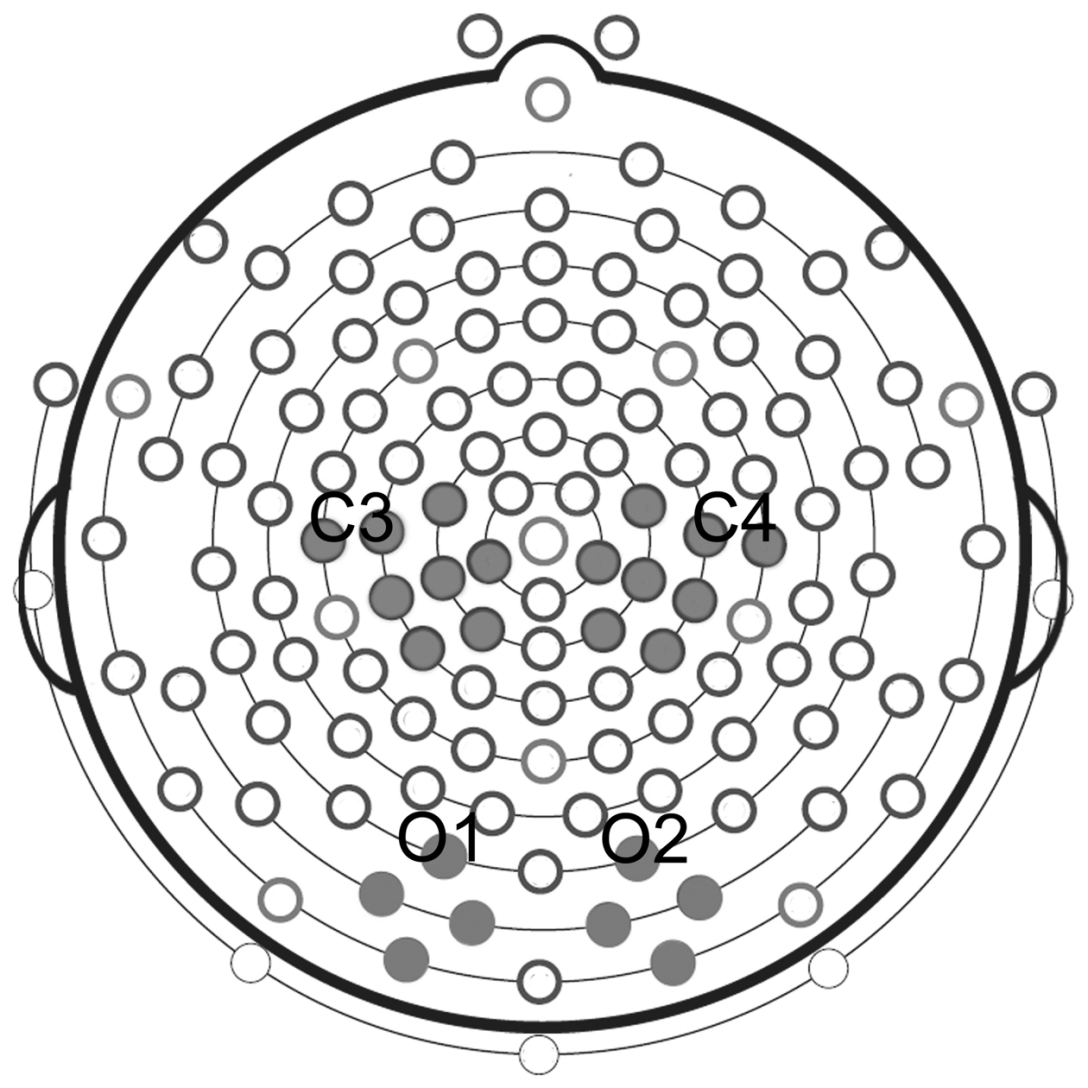
**Clusters of electrodes selected for statistical analyses.** Analysis of mu rhythm was performed on two clusters of eight electrodes each, located around standard C3 and C4 sites. Analysis of posterior visual alpha was carried out on two clusters of four electrodes each, located around standard O1 and O2.

Statistical analyses were performed on four selected clusters, 2 clusters of 8 electrodes in each hemisphere located around standard C3 and C4 sites for mu rhythm measurements, and 2 clusters of 4 electrodes each located around standard O1 and O2 for the control analysis of posterior visual alpha (Figure 2).

### Statistical analysis

For each participant alpha-frequency band was selected in the range of 8–14 Hz. For the following statistical analyses, the frequency-power in this 6 Hz range was extracted. The results of the wavelet transformation were used for statistical analysis. The data were extracted from two different time intervals: 500 ms of baseline; and from 250 to 750 ms after the appearance of the stimulus. The time interval selected as baseline for statistical analyses was from 2250 to 2750 after baseline onset. Please note that in order to avoid edge effects, the two time intervals (stimuli presentation and baseline) extracted for statistical analyses lasted 500 ms and were selected inside the larger window of 1000 ms used for wavelet transformation.

In order to assess mu rhythm suppression during visual presentation of the stimuli with respect to baseline, we performed a repeated measures 2 × 2 × 3 ANOVA (*p* ≤ 0.05) with Hemisphere (left vs. right), Familiarity (Familiar vs. Unfamiliar), and Condition (Baseline, Original, and Control conditions) as main factors.

In order to control for the individual variability in absolute EEG baseline power and to normalize the data, the amplitude squared (μv^2^) was transformed as the Log Ratio between the experimental conditions relative to the baseline power. Negative values represent a decrease of the mu rhythm power, positive values an increase of it. The log ratio of the frequency-power was calculated between the 500 ms of Baseline and the 500 ms of presentation of the Original and Control stimuli, respectively. A 2 × 2 × 2 repeated measures ANOVA (*p* ≤ 0.05) was performed with 2 levels of Hemisphere (left vs. right), 2 levels of Familiarity (Familiar vs. Unfamiliar), and 2 levels of Condition (Original vs. Control).

*Post-hoc* analyses (Newman–Keuls, test *p* ≤ 0.05) were applied on all significant main factors and interactions resulted from the ANOVAs.

In order to investigate the possible modulation of the beta component during the observation of the two categories of stimuli, two different Fast Fourier Transforms in the 8–14 Hz (mu) and 18–24 Hz (beta) ranges were computed on the entire head at group level. This analysis was performed on the same time window used for wavelet transformation (from 250 to 750 ms after the appearance of the stimulus) for both experimental conditions. Furthermore, we examined the beta-frequency band selected in the range of 18–24 Hz performing the wavelet transformation following the same steps of the analysis executed for the mu rhythm. The amplitude squared (μv^2^) of the beta power was transformed as the Log Ratio between the experimental conditions relative to the baseline power. The log ratio values of the beta frequency range were entered in a 2 × 2 × 2 repeated measures ANOVA with 2 levels of Rhythm (mu vs. beta), 2 levels of Hemisphere (left vs. right), and 2 levels of Condition (Original vs. Control).

In order to exclude that the mu rhythm recorded from the central areas was affected by the posterior alpha activity, data resulting from the wavelet transformation were extracted from two clusters of 4 electrodes each around O1 and O2 in the same two time intervals used for the central electrode analyses. The data extracted from occipital electrodes have been entered in a repeated measures 2 × 3 ANOVA (*p* ≤ 0.05) with the main factors of Hemisphere (left vs. right), and Condition (Baseline, Original, and Control stimuli).

The averages of the score that each participant gave to the “Amount of movement,” the “Aesthetic value,” and the “Artistic nature” of the 3 Original and the 3 Control stimuli were used to perform three different repeated measures ANOVAs (*p* ≤ 0.05) with 2 levels of Familiarity (Familiar vs. Unfamiliar) and two levels of Condition (Original vs. Control). *Post-hoc* analysis (Newman–Keuls, test *p* ≤ 0.05) was applied on all significant main factors and interactions.

## EMG

### Participants

Twelve healthy volunteers (6 males, 6 females, mean age 28.4 years; SD ± 5.9) participated in the experiment. The participants involved in the EMG recordings belonged to a different group from those already involved in the EEG experiments in order to avoid any training and/or experience effect. Written informed consent was obtained from all participants before entering the study. The Ethical Committee of the University of Parma approved the study.

### EMG recordings

In order to measure hand and arm EMG activation, Ag/AgCl surface electrodes (diameter 3 mm) were attached bipolarly over the right *First Dorsal Interosseous* (*FDI*) and right *Biceps* muscles. Continuous electromyography (EMG) recordings from both muscles were simultaneously acquired with a CED Micro 1401 analog-to-digital converting unit (Cambridge Electronic Design, Cambridge, UK). The EMG signal was amplified (1000×) digitized (sampling rate: 1 kHz) and stored on a computer for off-line analysis.

### EMG data analysis

Off-line, data were submitted to a 50–500 Hz band-pass filter to reduce movement-related artifacts and environmental noise, and have been full-wave rectified. Data were then visually inspected, and trials with remaining artifacts were excluded from further statistical analyses. For each participant and trial, the averaged EMG responses of the two muscles were subdivided in 4 time-bins of 250 ms each. For each participant and trial four time epochs of 250 ms preceding the appearance of the fixation cross have been used as baseline epochs for normalization of the EMG activity. Each epoch was normalized dividing it by the corresponding time-bin of the baseline.

### Statistical analysis

Emg data were entered into a 2 (Condition: Original and Control stimuli) × 2 (Muscles: * FDI* and *Biceps*) × 4 (Epochs: 0–250 ms, 250–500 ms, 500–750 ms, 750–1000 ms) repeated measures ANOVA (*p* < 0.05.), with Condition, Muscle, and Epochs as the within-subjects factors and Familiarity (Familiar vs. Unfamiliar) as between-subjects factor.

## Results

Repeated measures ANOVA was performed in order to assess mu rhythm suppression during observation of Original stimuli with respect to Control stimuli and to the baseline. The results showed a significant main effect of Condition [*F*_(2, 24)_ = 6.095; MS = 0.602; *p* < 0.01] regardless of Hemisphere and Familiarity [Emisphere × Condition × Familiarity: *F*_(2, 24)_ = 0.948; MS = 0.051; *p* > 0.1]. The comparisons among the three conditions showed that the squared amplitude of mu rhythm was significantly different from the Baseline only during the observation of Original artworks (*p* < 0.01), and that the observation of Control stimuli was significantly less effective (*p* < 0.05) in mu rhythm suppression than Original stimuli.

**Figure 3:**
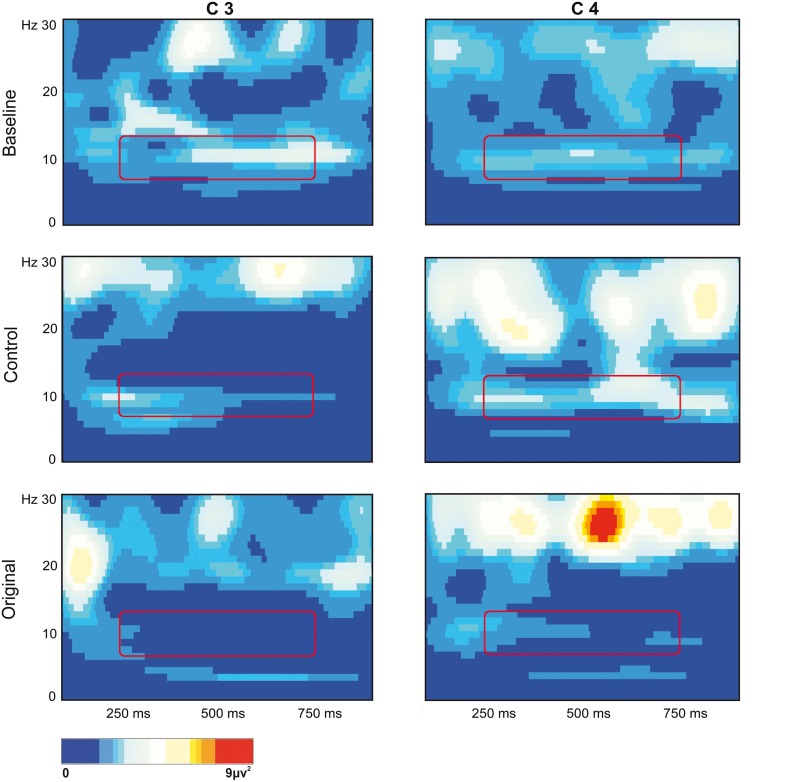
**EEG time-frequency spectrum of one participant extracted from C3 and C4 sites in the two experimental Conditions and in the Baseline.** A continuous wavelet transformation was performed for the frequencies from 1 to 30 Hz in a time window of 1 s, which corresponds to the total duration of stimulus presentation. Red color indicates increased mu rhythm squared amplitude, while blue color indicates decreased mu rhythm squared amplitude in a given frequency band (see scale bar). The rectangles added to each panel highlight the 7 Hz wide frequency band (8–14 Hz) and the time window (250–750 ms) used for statistical analysis.

The stronger mu rhythm suppression during observation of Original stimuli with respect to Control stimuli and to the Baseline is well exemplified in Figure 3 illustrating the EEG time-frequency spectrum of one participant extracted from C3 and C4 sites in the two experimental conditions and in the Baseline.

**Figure 4:**
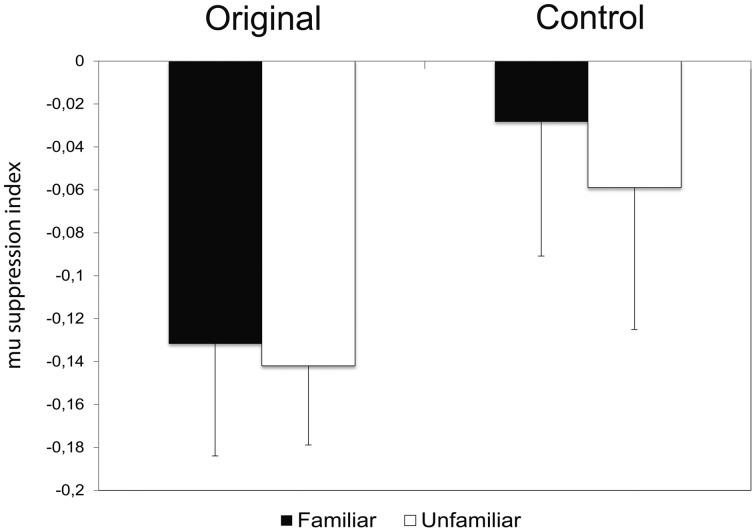
**Amplitude-squared of mu rhythm expressed as Log Ratio in Control and Original conditions.** The amplitude squared (μv^2^) was transformed as the Log Ratio between the experimental conditions relative to the Baseline power. Negative values represent a decrease of the mu rhythm power, positive values an increase of it. Plots represent the interaction Condition × Familiarity (*p* > 0.5) resulted from the ANOVA performed on mu rhythm expressed as Log Ratio.

The repeated measures ANOVA performed on the log ratio of the frequency-power confirmed the previous results, showing only a significant effect of Condition [*F*_(1, 12)_ = 6.621; MS = 0.034; *p* < 0.05]. The comparison between the two conditions demonstrated a stronger mu rhythm suppression (*p* < 0.05) during observation of the original artworks (Figure 4). The amount of mu rhythm suppression was higher when observing original artworks than control stimuli, again, regardless of participants' familiarity with the original artworks [Condition × Familiarity: *F*_(1, 12)_ = 0.078; MS = 0.000; *p* > 0.5; Emisphere × Condition × Familiarity: *F*_(2, 24)_ = 0.948; MS =0.001; *p* > 0.5].

**Figure 5:**
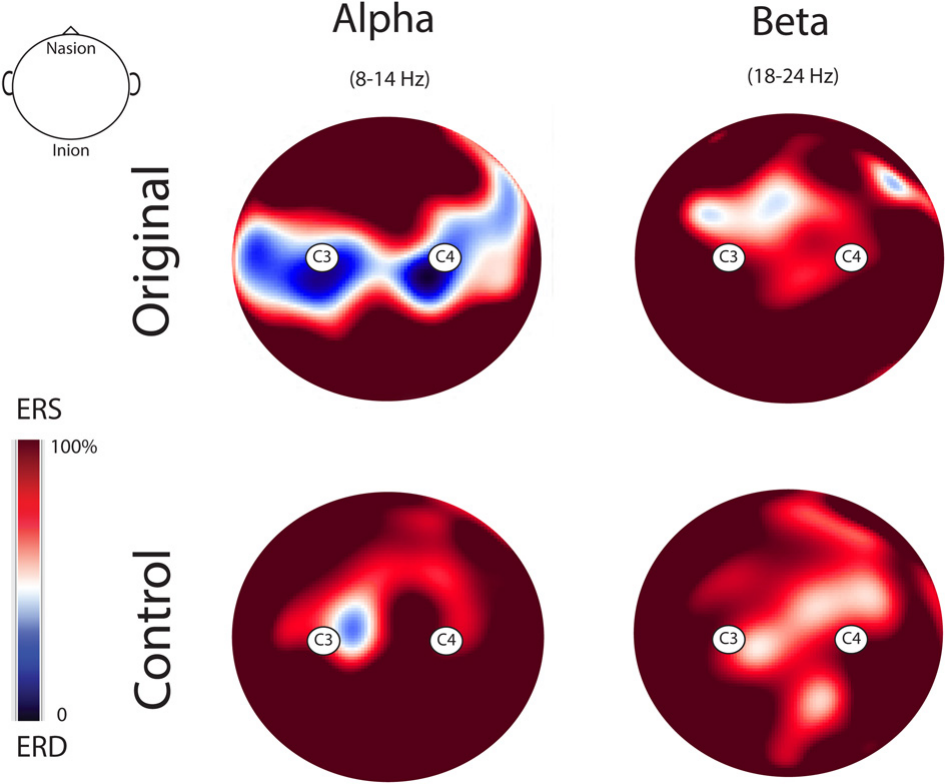
**Topo maps of the fast Fourier transforms performed on the alpha and beta ranges in both experimental conditions.** Mu suppression was bilateral and stronger during the observation of the Original Artworks compared with the observation of Control stimuli. Beta range modulation was absent in both experimental conditions.

Figure 5 illustrates the topo maps of the fast Fourier transforms performed on the alpha and beta ranges in both experimental conditions. Mu suppression was bilateral and mainly localized over the sensorimotor areas. Furthermore, mu suppression was much stronger during the observation of the Original Artworks compared with the observation of Control stimuli. Differently from mu rhythm, beta range modulation was absent in both experimental conditions. The confirmation of the lack of beta modulation during the observation of both categories of stimuli came from the results of the statistical analysis performed on the log ratio of the beta range (18–24 Hz). The results of the ANOVA showed a significant main effect of Rhythm [*F*_(1, 13)_ = 26.322; MS = 0.004; *p* < 0.00] and a significant interaction Rhythm × Condition [*F*_(1, 13)_ = 4.39; MS =0.013; *p* = 0.05]. Newman–Keuls *post-hoc* comparisons applied to main effect of Rhythm showed that alpha suppression was significantly stronger than beta (*p* < 0.00). Moreover, *post-hoc* applied to the significant interaction Rhythm × Condition indicated that while mu was modulated by conditions, as previously demonstrated, with the strongest suppression during the observation of Original Artworks (*p* < 0.00), beta rhythm remained unchanged across conditions (*p* > 0.8).

**Figure 6:**
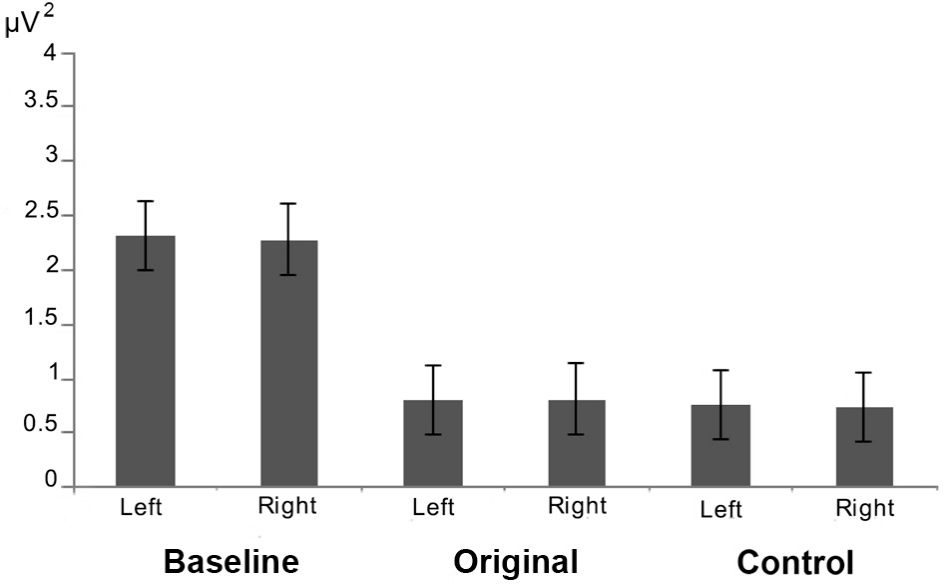
**Results of the analysis performed on the alpha power extracted from occipital electrodes.** Plots show that that the observation of the two different categories of stimuli did not evoke any difference in the posterior visual alpha modulation in both hemispheres.

The results of the analysis performed on the alpha power extracted from 2 clusters of 4 electrodes each around O1 and O2 showed only a significant main effect of Condition [*F*_(2, 26)_ = 8.673; MS = 2.696; *p* < 0.005] (see Figure 6). The further analysis with the Newman–Keuls test, however, revealed that the observation of the two different categories of stimuli did not evoke any difference in alpha rhythm squared amplitude (*p* > 0.1) while both of them were different from the baseline (*p*_s_< 0.05).

**Figure 7:**
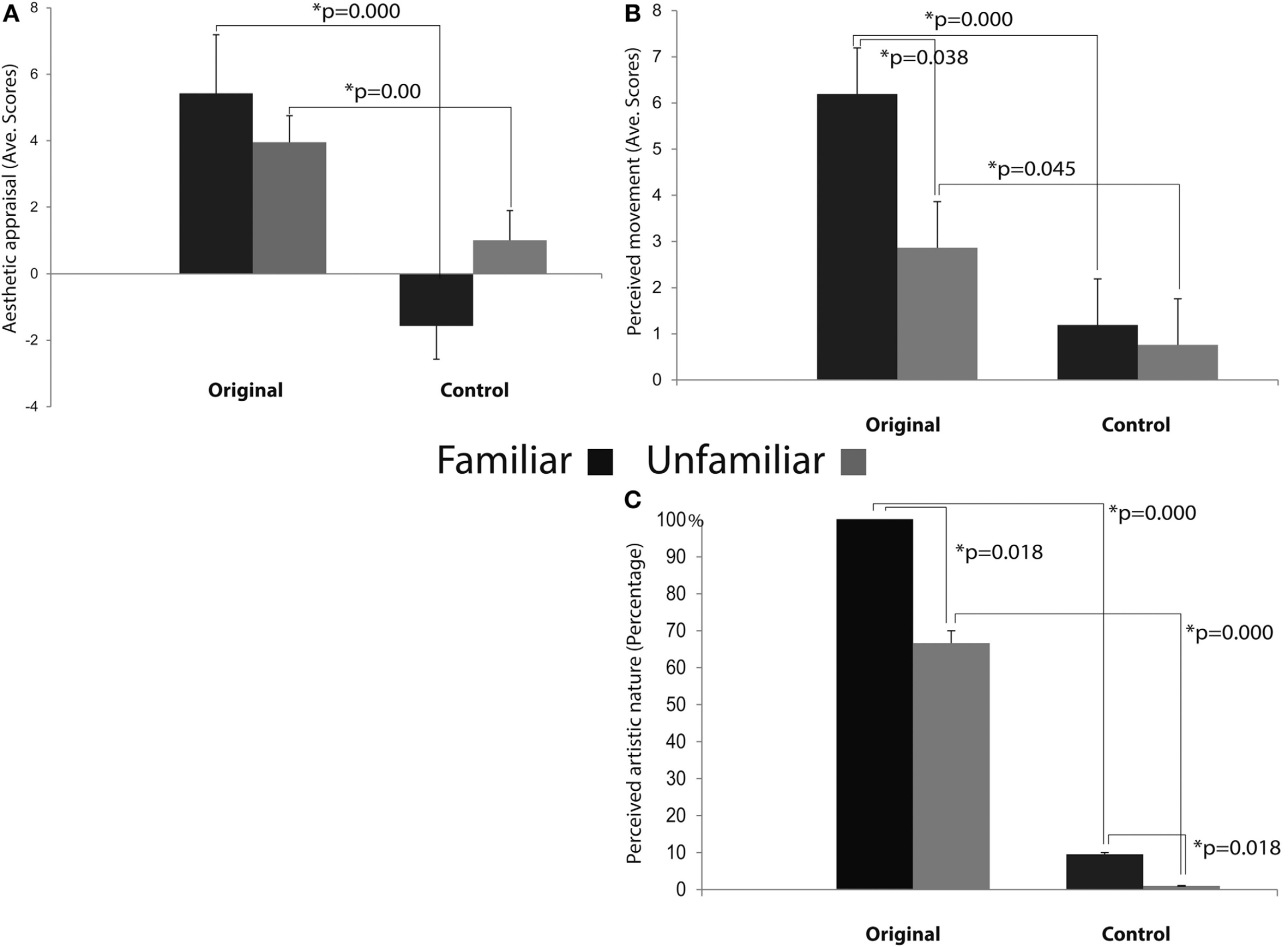
**Results of the ANOVAs performed on the aesthetic appraisal (A), amount of perceived movement (B), and artistic nature (C) in both groups of participants.** Plots of panels **(A)** and **(B)** represent the significant Condition × Familiarity interaction. The interaction was not significant in panel **(C)**.

The results of the repeated measures ANOVA performed on participants' “Aesthetic appraisal” (see Figure 7A) showed a significant main effect of Condition [*F*_(1, 12)_ = 55.977; MS = 173.33; *p* < 0.00] accompanied by a significant interaction Condition × Familiarity [*F*_(1, 12)_ = 9.258; MS = 26.67; *p* < 0.05]. The Newman–Keuls comparisons done on the main effect of Condition showed that participants' aesthetic appraisal was higher for original artworks than for control stimuli (*p* < 0.001). The investigation of the significant interaction Condition × Familiarity showed that in both groups of participants stimuli familiarity did not affect aesthetic appraisal of Original artworks and Control stimuli (*p*_s_> 0.1). However, both groups of participants gave a higher aesthetic appraisal when observing Original artworks than Control stimuli (*p*_s_ < 0.01).

The analysis of the score that participants gave to the “Amount of perceived movement” of the Original and Control stimuli (see Figure 7B) showed a significant main effect of Condition [*F*_(1, 12)_ = 28.683; MS = 88.099; *p* < 0.001] and a significant interaction Condition × Familiarity [*F*_(1, 12)_ = 4.807; MS = 14.765; *p* < 0.05]. The *post-hoc* comparisons, performed on the significant main effect of Condition, clearly showed that the amount of perceived movement scored by participants was significantly higher when they saw the original artworks if compared to the observation of the Control images (*p* < 0.005). When the comparisons were made on the significant interaction Condition × Familiarity, the amount of movement perceived in Original stimuli was higher among Familiar participants when compared to the Unfamiliar group (*p* < 0.05). In addition, movement perception for original artworks, compared with Control stimuli, was higher in both groups of participants, regardless of their being familiar (*p* < 0.005) or unfamiliar with them (*p* < 0.05).

Mean scores of the “Artistic nature” questionnaire were: Familiar 100% SE ± 0 for Original stimuli and 9.52% SE ± 4.76 for Control stimuli; Unfamiliar 66.66% SE ± 4.76 for Original stimuli and 0% SE ± 0 for Control stimuli (see Figure 7C). The statistical analysis showed that the main effects of Condition [*F*_(1, 12)_ = 99.00; MS = 4.321; *p* < 0.001] and Familiarity [*F*_(1, 12)_ = 7.363; MS = 0.321; *p* < 0.05] were significant, while the interaction between them was not [*F*_(1, 12)_ = 2.272; MS = 0.099; *p* > 0.1] (Figure 7C). *Post-hoc* comparisons were applied to the two significant main effects. Original artworks, when compared with Control images, were considered more often as real artworks (*p* < 0.005) by both groups of participants. Familiar participants gave more affirmative responses than the Unfamiliar group (*p* < 0.05). The lack of significance of the interaction between factors indicates that both groups of participants were more prone to judge the Original stimuli as real art works.

None of the main factors or interactions resulted from the ANOVA performed on the EMG data were statistically significant (all *p*_s_ > 0.05).

**Figure 8:**
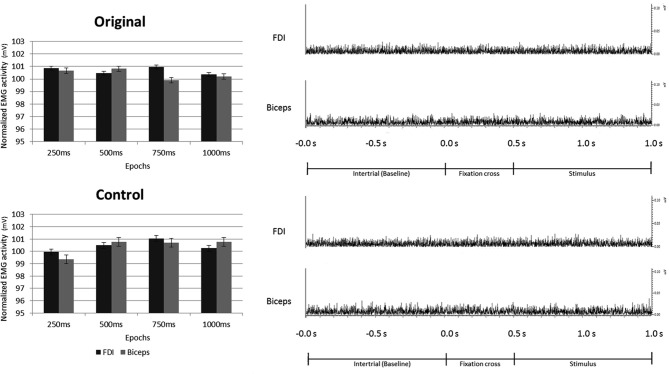
**Electromyography recorded from right *First Dorsal Interosseous* and *Biceps* muscles during observation of Original and Control stimuli.** Plots presented in the left part of the figure illustrate the results of the statistical analysis indicating that the observation of both, Original and Control stimuli did not evoke any EMG activation of the right hand and arm. The right part of the figure shows an example of the EMG activity recorded in one participant from both muscles, during one trial of both experimental conditions.

These results clearly demonstrate that the observation of both Original and Control stimuli did not evoke any EMG activation of participants' right hand (*FDI*) and arm (*Biceps*) muscles (see Figure 8).

## Discussion

The present results show that in spite of the similarity between the digitized versions of the Original artworks and the Control stimuli, only the former evoke cortical motor activation in the beholder's brain, as exemplified by mu rhythm suppression recorded from two selected clusters of 8 electrodes in each hemisphere located around standard C3 and C4 sites. The same clusters were used in previous studies demonstrating mu rhythm suppression during the observation of hand motor acts (Muthukumaraswamy et al., 2004; Muthukumaraswamy and Johnson, 2004a,b; Streltsova et al. 2010).

The motor activation induced by the observation of Original art works is confirmed by the results of the analysis performed on occipital clusters of electrodes. Such analysis clearly shows that mu rhythm suppression does not reflect posterior alpha activity, since the observation of the two different categories of stimuli did not evoke any difference in occipital visual alpha.

The results of the analyses performed on beta rhythm did not reveal any significant modulation across conditions. Some authors argued that the beta component would predominantly originate from motor cortex whereas the alpha component would mainly arise from postcentral sensory cortex (Hari and Salmelin, 1997). However, most recent studies aiming at correlating fMRI BOLD signal with EEG signal during motor tasks demonstrated a negative significant correlation between alpha ERD and BOLD signal not only on sensorimotor cortex, but also and most interestingly on premotor cortices (Arnstein et al., 2011; Yuan et al., 2011; Mizuhara, 2012). Furthermore, it should be added that even granting that sensorimotor alpha ERD originates more posteriorly than beta desynchronization, recent data in humans emphasize that the postcentral cortex, and in particular BA 2, must be considered integral part of the mirror neuron system (see Keysers et al., 2010; Arnstein et al., 2011).

Why should the observation of a static image like a cut in a canvas activate the observer's motor cortex? It is well known that the observation of specific categories of visual stimuli induces the activation of the observer's cortical motor system (see Gallese and Sinigaglia, 2011a). In particular, it has been shown both in monkeys and humans that the observation of motor acts and gestures activates their motor representations in the observers' brain (for review, see Rizzolatti and Sinigaglia, 2010).

More specifically, several EEG studies have shown mu rhythm suppression during the observation of different types of grasping (Muthukumaraswamy et al., 2004; Muthukumaraswamy and Johnson, 2004a; Perry and Bentin, 2009; Streltsova et al. 2010), meaningless gestures (Babiloni et al., 2002; Streltsova et al. 2010) and sequential finger movements (Calmels et al., 2006). It is interesting to note that such motor activation can also be induced by the observation of *static images* portraying actions (Johnson-Frey et al., 2003; Mado-Proverbio et al., 2009).

The present results extend the notion of cortical motor activation during the observation of visual stimuli to the observation of abstract static images. Our interpretation is that the observation of the cuts on canvases activates the motor representation of the same gesture in the brain of beholders. In contrast, Control stimuli, because of their lack of implicit dynamicity, as shown by participants' score on the perceived amount of movement, are merely processed as images that are not the manifest consequence of someone's gesture. Beside the observation of dynamic or static motor acts, also the observation of the static consequences of a motor act is capable of activating its motor representation in the observer's brain. Our data demonstrate that viewing cuts and viewing lines differently impact on beholders' cortical motor system. Of course, we neither claim that the perceived esthetic status of the work is a necessary condition for the evocation of such cortical motor activation, nor that cortical motor activation is a necessary component of esthetic ranking. What we have found, however, is that such cortical motor activation is a component of what happens in beholders' brain during the observation of art works such as those of Lucio Fontana used as stimuli in the present study. Furthermore, cortical motor activation occurs in spite of the lack of activation of beholders' contralateral hand and arm muscles, as demonstrated by the EMG recordings. For these reasons such cortical motor activation may be regarded as a form of embodied simulation (see Gallese, 2003, 2005; Gallese and Sinigaglia, 2011b) understood as a functional mechanism characterized by the reuse of motor representations when observing others' actions or, as in the present case, the [visual] results of such actions.

To the best of our knowledge this is the first study to explicitly address the relationship between brain activity during the observation of works of art and beholders' visual familiarity with them. Interestingly enough, we found that the cortical motor activation induced by the observation of Fontana's art works did not depend at all on previous exposure to them, as demonstrated by the lack of significance of the factor Familiarity in the mu rhythm suppression analyses.

The empirical investigation of the basic neural mechanisms underpinning the responses to art is a complex issue. There is great heterogeneity across results from investigations trying to clarify the neural correlates associated with the perception of visual art (for review, see Di Dio and Gallese, 2009; Gallese and Di Dio, 2012). The perception of visual art works begins with the visual analysis of the stimulus, which then undergoes further processing. This progression may lead to an “aesthetic experience” likely based on biological and embodied mechanisms that are modulated by factors such as the context, individuals' interest in the artwork, prior knowledge, and familiarity. We suggest that beholders' cortical motor activation constitutes an important component of the perception of abstract art works of the kind shown here—as well, we would propose, in the case of more explicit representations of movement in pictures.

Behavioral results obtained after EEG data collection showed that participants' aesthetic appraisal was higher for Original artworks than for Control stimuli, independently of their visual familiarity with them. In both groups of participants, Original art works evoked significantly higher scores for aesthetic appraisal than control stimuli. This result shows that prior familiarity with an image is not required to affect the explicit aesthetic appraisal of the perceived object. Behavioral results also showed that Original art works by Fontana were rated as looking more dynamic than Control stimuli in both groups of participants, although in this case visual familiarity with Original art works influenced the amount of perceived movement. In principle, it is possible that participants familiar with Fontana's work also knew how the image was produced by the artist, and may thus have been influenced in their rating by their motor imagery of the artist's cutting gesture. However, the mu rhythm suppression results clearly contradict this hypothesis, since cortical motor activation was not influenced by participants' familiarity with the stimuli.

Our results provide the first evidence of the involvement of the cortical motor system in viewing static images of abstract art, even when devoid of any explicit representation of movement, and independent of visual familiarity. While the perceived esthetic status of these works is probably not a necessary requisite to evoke cortical motor activation, we do take such activation to be an important component of the perception of such works of art. The present results lend empirical support to the role of motor simulation in the observation of abstract art (Freedberg and Gallese, 2007; Di Dio and Gallese, 2009).

### Conflict of interest statement

The authors declare that the research was conducted in the absence of any commercial or financial relationships that could be construed as a potential conflict of interest.
